# Active case detection of malaria in pregnancy using loop-mediated amplification (LAMP): a pilot outcomes study in South West Ethiopia

**DOI:** 10.1186/s12936-020-03380-9

**Published:** 2020-08-27

**Authors:** Guluma Tadesse, Claire Kamaliddin, Cody Doolan, Ranmalee Amarasekara, Ruth Legese, Abu Naser Mohon, James Cheaveau, Delenasaw Yewhalaw, Dylan R. Pillai

**Affiliations:** 1 Institute of Health, School of Medical Laboratory Sciences, Jimma University, Jimma, Ethiopia; 2 Cumming School of Medicine, Department of Pathology & Laboratory Medicine, Diagnostic & Scientific Centre, The University of Calgary, Room 1W-416, 9-3535 Research Road NW, Calgary, AB T2L 2K8 Canada; 3 Cumming School of Medicine, Department of Microbiology, Immunology, and Infectious Diseases, The University of Calgary, Calgary, Canada; 4 Tropical and Infectious Diseases Research Center, Jimma University, Jimma, Ethiopia

**Keywords:** Malaria, Asymptomatic infections, Pregnancy, LAMP, Low birth weight, Anemia, Ethiopia

## Abstract

**Background:**

125 million women are pregnant each year in malaria endemic areas and are, therefore, at risk of Malaria in Pregnancy (MiP). MiP is the direct consequence of *Plasmodium* infection during pregnancy. The sequestration of *Plasmodium falciparum* parasites in the placenta adversely affects fetal development and impacts newborn birth weight. Importantly, women presenting with MiP commonly develop anaemia. In Ethiopia, the Ministry of Health recommends screening symptomatic women only at antenatal care visits with no formal intermittent preventive therapy. Since MiP can display low-level parasitaemia, current tests which include microscopy and RDT are challenged to detect these cases. Loop mediated isothermal Amplification (LAMP) technology is a highly sensitive technique for DNA detection and is field compatible. This study aims to evaluate the impact of active malaria case detection during pregnancy using LAMP technology in terms of birth outcomes.

**Methods:**

A longitudinal study was conducted in two health centres of the Kafa zone, South West Ethiopia. Both symptomatic and asymptomatic pregnant women were enrolled in the first or second trimester and allocated to either Standard of Care (SOC—microscopy and RDT) or LAMP (LAMP, microscopy and RDT). Women completed at least three visits prior to delivery, and the patient was referred for treatment if *Plasmodium* infection was detected by any of the testing methods. The primary outcome was to measure absolute birth weight, proportion of low birth weight, and maternal/neonatal haemoglobin in each arm. Secondary outcomes were to assess the performance of microscopy and RDT versus LAMP conducted in the field.

**Results:**

One hundred and ninety-nine women were included and assigned to either LAMP or SOC. Six were lost to follow up. In this cohort, 66.8% of women did not display any clinical symptoms and 70.9% were multi-parous. A reduced proportion of low birth weight newborns was observed in the LAMP group (0%) compared to standard of care (14%) (*p *<0.001). Improved neonatal haemoglobin was observed in the LAMP (13.1 g/dL) versus the SOC (12.8 g/dL) (p = 0.024) arm. RDT and microscopy had an analytical sensitivity of 66.7% and 55.6% compared to LAMP as a reference standard.

**Conclusions:**

These results support the use of highly sensitive tools for rapid on-site active case detection of MiP which may improve birth outcomes in the absence of IPT. However, further large-scale studies are required to confirm this finding.

## Background

Malaria in pregnancy (MiP) is the consequence of *Plasmodium* infection during pregnancy, and potentially leads to severe disease. MiP results in adverse birth outcomes such as low birth weight (LBW) (defined by a birth weight < 2500 g), small for gestational age fetus (< 10th percentile), pre-term birth (< 37 weeks of gestational age) and stillbirth. *Plasmodium* infection and subsequent sequestration of infected erythrocytes in the placenta restricts maternal blood supply, oxygen, and nutrients necessary for normal fetal development [1]. Maternal anaemia is another complication and results from peripheral destruction of infected erythrocytes during *Plasmodium* asexual multiplication. Nearly 125 million women are pregnant each year in malaria endemic areas and are, therefore, at risk of MiP [2]. In malaria endemic areas, it is estimated that 20% of stillbirth and 11% of all newborn deaths are direct consequences of MiP [3]. Both *Plasmodium falciparum* and *Plasmodium vivax* induce adverse pregnancy outcomes. *Plasmodium falciparum* pathogenicity in MiP is well described [4, 5] while the pathophysiology of *P. vivax* infection during pregnancy needs yet to be elucidated [6, 7].

To prevent the overall clinical and economic burden of MiP, vector protection measures, such as long-lasting insecticide-treated nets are supported. Most countries in sub-Saharan Africa adopt an intermitted preventive treatment (IPT) strategy as per the World Health Organization (WHO) recommendations [8]. IPT consists of mass administration of sulfadoxine-pyrimethamine to pregnant women, starting in the second semester of pregnancy at each antenatal care visit. The use of IPT has demonstrated a positive impact on LBW prevalence and neonatal mortality, by reducing the malaria episodes and the parasitic burden in pregnant women [9, 10]. However, the use of IPT presents several limitations. First, if the treatment is not administered completely or with poor compliance, IPT contributes to the spread of drug resistant parasites [11, 12]. Second, gametocytes are detected in pregnant women following the administration of IPT [13], which is one of the contributors to malaria transmission. Third, depending on country resources, political stability and strategies for IPT implementation, the coverage of IPT can remain low [14] and IPT implementation may be compromised by medication stock-outs. The WHO estimated that as of 2018, coverage rates for IPT at each antenatal visit were respectively 60%, 49% and 31% [15]. Fourth, the timing of IPT administration does not allow impact of treatment on early pregnancy infections, which can occur as early as 13 weeks of pregnancy [16]. Early pregnancy infections (before 20 weeks of pregnancy) have an impact on fetal growth and are associated with a lower birth weight at delivery [17, 18].

The alternative to IPT strategies is active case detection when women are screened at each antenatal visit for parasite carriage and treated only if *Plasmodium* spp. are detected. To conduct efficient active case detection, there is a necessity for highly sensitive and affordable point of care (POC) malaria diagnostic tests. Conventional malaria diagnostic methods such as stained blood smear and rapid diagnostic tests (RDT) offer limited sensitivity, with limits of detection (LOD) > 50 parasites per μL. RDT sensitivity is a limitation for MiP active case detection as shown by a clinical trial in Kenya [19]. High sensitive testing with molecular tools such as PCR showed an increase in MiP detection, with 2–2.5-fold more cases detected [20, 21]. Since asymptomatic *P. falciparum* infection during pregnancy is associated with increased maternal anaemia [21], there is a need to evaluate the impact and feasibility of analytically sensitive POC malaria diagnostic. Loop mediated isothermal amplification (LAMP) has excellent analytical sensitivity, can be performed with minimum equipment requirements, and provides a rapid result within 45 min [22, 23]. LAMP is one of the isothermal molecular techniques compatible with POC settings. A pilot study was conducted to evaluate active case detection of MiP using LAMP compared to conventional techniques (microscopy and RDT) only. To do so, a prospective outcomes-based study was conducted in two health centres of the Kafa zone, South West Ethiopia.

## Methods

### Study population, design, and ethical approvals

**Fig. 1:**
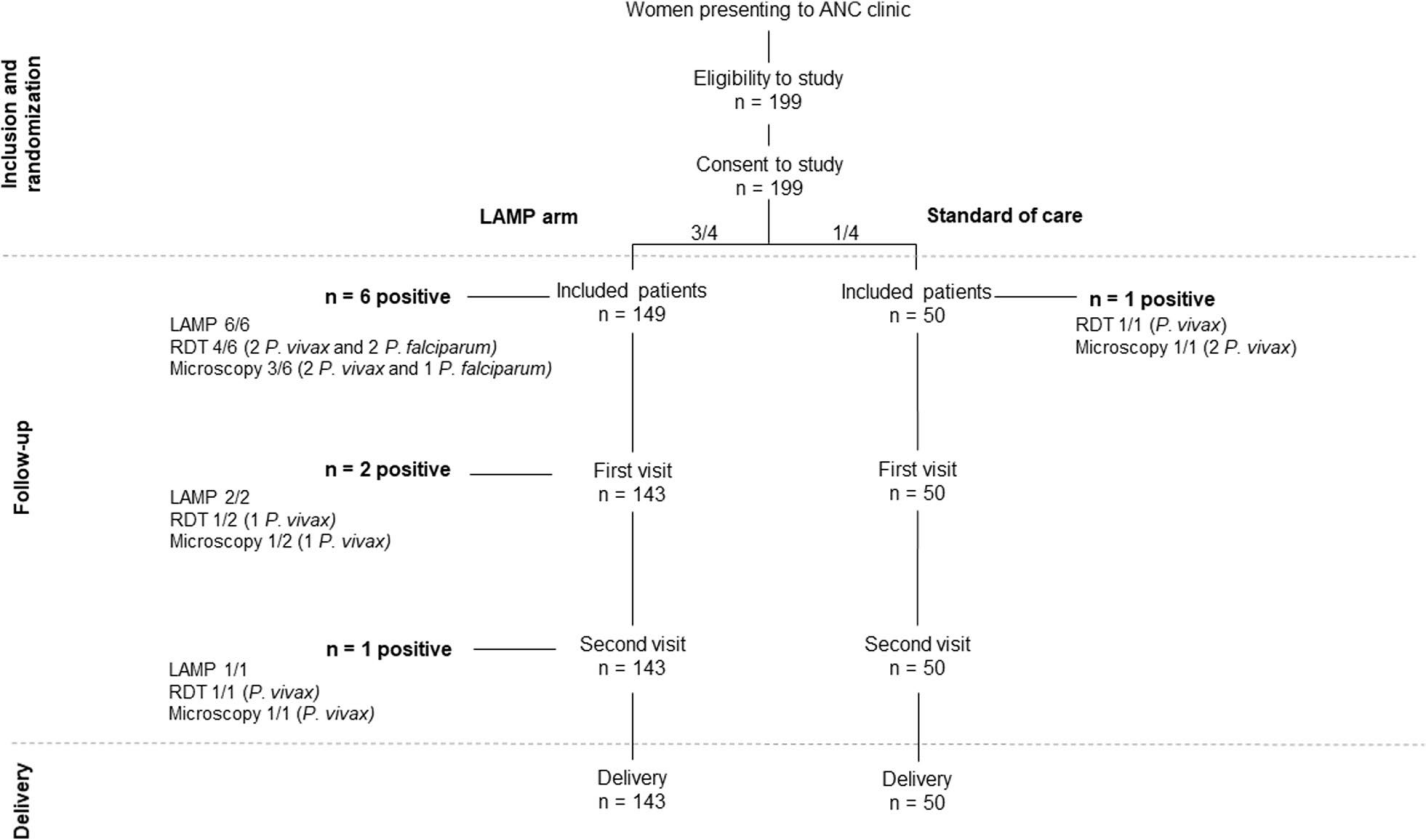
Flowchart depicting enrolment and follow-up of pregnant women at Gimbo and Gojeb Health Centres, Kafa Zone, SW Ethiopia

The study was conducted at Gimbo and Gojeb Health Centres, Kafa Zone, SNNP Regional State, Ethiopia. The zone sits at an altitude between 500 and 3500 m above sea level. Both *P. vivax* and *P. falciparum* are endemic with a seasonal transmission which peaks from September to December. A retrospective study in the Jimma area from September 2007 to August, 2017 showed the prevalence dropping from 27.9% to 0.62% between 2007 and 2016 [24]. Pregnant women in their first or second trimester of pregnancy attending ANC were included in the study from October 2018 to January 2019. Women presenting with severe malaria symptoms according to WHO guidelines and/or who received anti-malarial drugs in the past four weeks were excluded. Included women were allocated to the standard of care (SOC) arm (1/4) where women were tested by microscopy and RDT, or the LAMP arm (3/4) where women were tested by LAMP, microscopy and RDT (Fig. 1). Women were assigned consecutively between the two study arms during the inclusion visit as they presented in the aforementioned ratio. Women were deemed symptomatic based on self-report of febrile episodes in the past seven days or upon questioning and medical examination at time of enrolment. Women were required to make at least three ANC visits (including enrolment) in addition to the delivery. Testing for haemoglobin and malaria was performed at each visit. Women testing positive for malaria by microscopy/RDT and/or by LAMP technology depending on the study arm were referred to a medical officer for further work up and treatment according to the Federal Ministry of Health guidelines. The study was approved by the research ethics board at Jimma University (IHRPGD/3027/18) and University of Calgary (CHREB 17-1335).

### Laboratory and clinical testing at ANC visit

Women were tested for malaria (described later) and haemoglobin (CareStart biosensor, WellsBio, USA) was measured at each visit. Women with haemoglobin levels lower than 11.0 g/dL were considered anaemic. Anaemic patients were subcategorized as mild anaemia (Hb 10.0–10.9 g/dL), moderate anaemia (Hb 7.0–9.9 g/dL) and severe anaemia (Hb lower than 7 g/dL) following the WHO recommendation for pregnant women [25]. Whole blood was collected through venous puncture on EDTA anticoagulants tubes. Peripheral blood examination (slides) and RDT (CareStart™ HRP2/pLDH COMBO (Pf/Pan) detection kits [Accesbio, Korea]) were performed on all samples. Slides (thick and thin films) were air dried, fixed with methanol, and stained with 10% Giemsa. Slides were read at 100× oil immersion objective and considered negative after 2000 leucocytes were counted. Microscopy results were made by two independent medical technologists and a third blinded read was performed in case of discrepancy regarding positivity. Parasitaemia per microlitre were estimated using white blood cell (WBC) count or the average of 8000/μL if the WBC count was not available. In the LAMP arm, *Plasmodium* spp. DNA was additionally detected using a commercial CE-marked LAMP assay (Illumigene, Meridien Bioscience, Cincinnati, OH) as per manufacturer’s recommendations. Birth outcome and newborn related variables, were performed at the head-to-toes assessment. Fetal haemoglobin was measured using CareStart biosensor (WellsBio, USA) and newborn weight was assessed using standard issue Federal Ministry of Health scales before first feeding.

### Statistical analysis

All statistical analysis were conducted in R software [26]. Graphical outputs were prepared using ggplot2 package [27]. Proportions were compared using Chi-squared test and continuous variables with Student’s t test or Wilcoxon Mann–Whitney test.

## Results

### Demographic data on enrolled patients

One hundred and ninety-nine women were included in the study (99 in Gojeb centre and 100 in Gimbo centre). Women were allocated to the LAMP arm (73 in Gojeb and 76 in Gimbo) or the SOC arm (26 in Gojeb and 24 in Gimbo) using microscopy and/or RDT for malaria diagnosis. Patient characteristics at inclusion are shown in Table 1 and did not differ significantly between the two study arms. Overall, 193 women were followed until delivery (50/50 in the SOC arm and 143/149 in the LAMP arm). Six women (3%) were lost to follow up due to relocation, delivery at another facility, or logistical challenges.

**Table 1:** Description of the population at inclusion of the study

Characteristic	All (n = 199)	LAMP (n = 149)	SOC (n = 50)	*P*
Age, mean (SD), years	26.4 (4.6)	26.2 (4.6)	27.1(4.8)	0.224
Haemoglobin, median (IQR), g/dL	12.00 (1.25)	12.00 (1.20)	12.01 (1.30)	0.698
Parity, no. (%)				0.012
Primiparous	58 (29.1)	36 (24.2)	22 (44.0)	
Multiparous	141 (70.9)	113 (75.8)	28 (56.0)	
Gestational age, mean (SD), weeks	16.4 (5.8)	16.4 (5.8)	16.3 (5.7)	0.863
Educational level, no. (%)				0.794
Illiterate	78 (39.2)	60 (40.3)	18 (36.0)	
Read/write	84 (42.2)	64 (42.9)	20 (40.0)	
Primary school	23 (11.6)	16 (10.7)	7 (14.0)	
Secondary school	12 (6.0)	8 (5.4)	4 (8.0)	
College/above	2 (1.0)	1 (0.7)	1 (2.0)	
Occupation, no. (%)				0.209
Daily labourer	56 (28.1)	37 (24.8)	19 (38.0)	
Farmer	85 (42.7)	66 (44.3)	19 (38.0)	
Merchant	18 (9.1)	16 (10.7)	2 (4.0)	
Housewife	40 (20.1)	30 (20.1)	10 (20.0)	
Use of ITN, no. (%)				0.897
No usage	80 (40.2)	61 (40.9)	19 (38.0)	
Rare	83 (41.7)	62 (41.7)	21 (42.0)	
Systematic	36 (18.1)	26 (17.4)	10 (20.0)	
*Plasmodium* spp. prevalence*, %	4/199 (2.0)	3/149 (2.0)	1/50 (2.0)	0.801

Data are presented for all patients, as well as for each study arm. *P*-values are displayed for comparison between the two arms. Categorical variable were compared using Chi square test and continuous variables using Student’s t test

*IQR* interquartile range, *SD* standard deviation, *ITN* insecticide treated nets, *LAMP* loop mediated amplification, *SOC* standard of care

* At inclusion, estimation using stained thick blood smear

### Malaria prevalence and asymptomatic malaria at inclusion

**Fig. 2:**
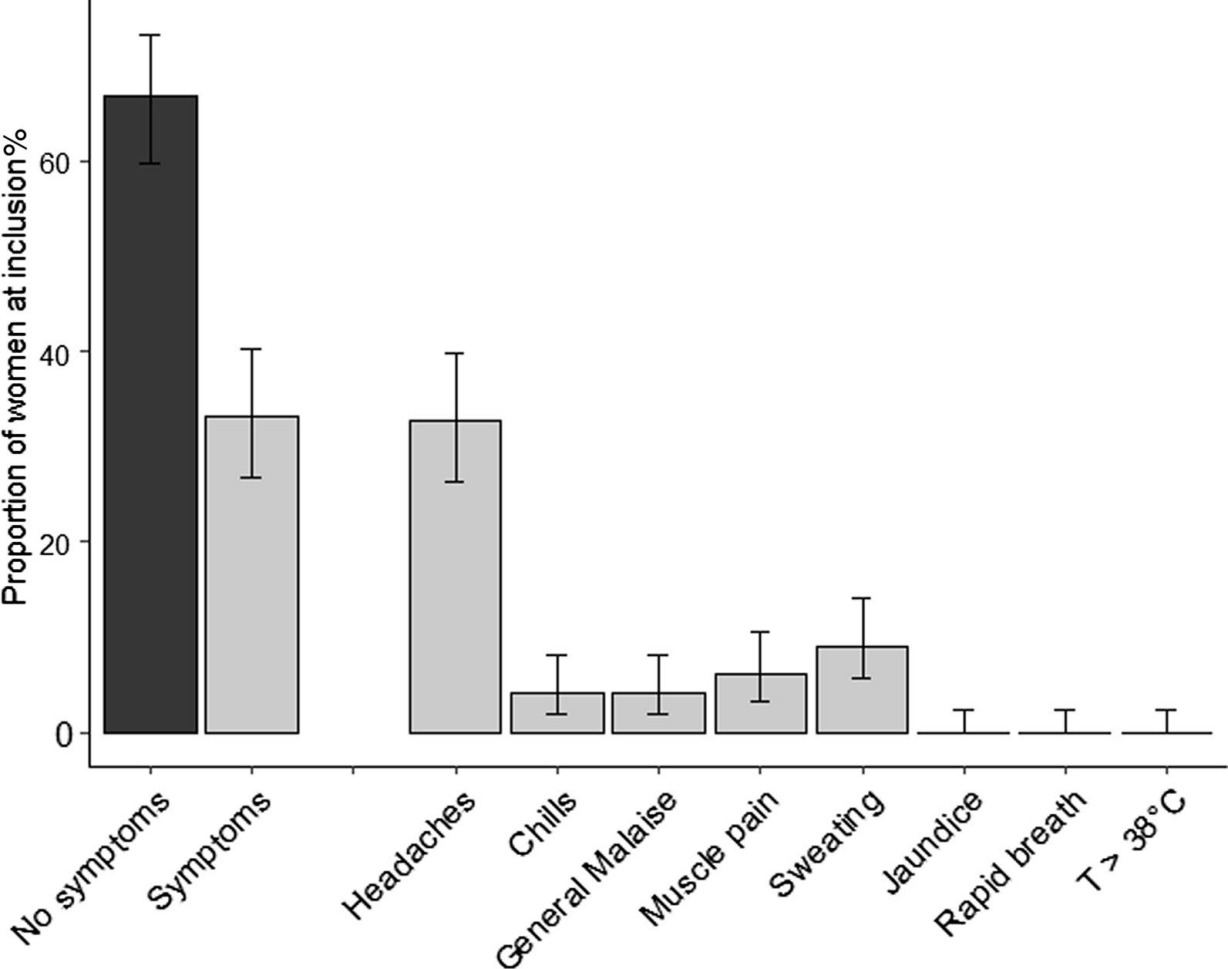
Characteristics of women at study inclusion (n = 193). Bars represent the proportion (%) of women with corresponding symptoms. Errors bars represent 95% confidence interval

At inclusion, 66.8% (CI_95%_ 59.8–73.3) of women did not display any clinical symptoms. Among women presenting with symptoms, headaches represented 32.7% (CI_95%_ 26.6–39.7), sweating 9.0% (CI_95%_ 5.6–14.1) and muscle pain 6.0% (CI_95%_ 3.3–10.5) (Fig. 2). Overall, malaria prevalence at diagnosis was 2.0% (n = 4/200) (CI_95%_ 0.7–5.4) using microscopy/RDT and 4.2% (n = 6/149) (CI_95%_ 1.7–8.9) using LAMP additionally at inclusion. Both *P. falciparum* and *P. vivax* were detected. Average parasitaemia estimated by microscopy was 882.7 p/µL (range 0.94–1150 p/µL) at inclusion.

### Malaria during pregnancy and birth weight

Women were followed up during pregnancy for a total of three visits (including enrolment) during pregnancy and a visit at delivery. In the SOC arm, no additional malaria case was diagnosed during follow-up. In the LAMP arm, 1.4% (CI_95%_ 0.2–5.4) were positive at the first visit and 0.7% (CI_95%_ 0.03–0.4) at the second follow-up visit. Cumulative prevalence of malaria was 2.0% (CI_95%_ 0.1–1.2) in the SOC arm and 6.2% (CI_95%_ 3.1–11.9) in the LAMP arm. At delivery, no adverse major event (preterm deliveries and stillbirth) was noticed in any women (Table 2). Average birth weight was 3.034 kg (Q1–Q3 [2.750–3.200] kg) for all newborn. Average birth weight was lower in the SOC arm than in the LAMP arm (2.965 vs. 3.059 kg) but the difference was not statistically significant (*p *= 0.148). Regarding the proportion of LBW, a significant difference between the standard of care group (14% of LBW) and the LAMP group (0%) (*p *<0.001) was observed.

**Table 2:** Birth outcomes related to the study

Characteristic	All (n = 193)	LAMP (n = 143)	SOC (n = 50)	*p*
Preterm birth (no, %)	0 (0)	0 (0)	0 (0)	N/A
Still birth (no, %)	0 (0)	0 (0)	0 (0)	N/A
Newborn haemoglobin (g/dL) (mean, Q1–Q3)	13.0 [12.7–13.6]	13.1 [12.8–13.7]	12.8 [12.0–13.4]	0.024
Maternal haemoglobin (g/dL) (mean, Q1–Q3)	11.5 [11.0–12.0]	11.5 [11.0–12.0]	11.6 [11.0–12.0]	0.771
Low birth weight (no, %)	7 (3.5)	0 (0)	7 (14)	< 0.001
Birth weight (kg) (mean, Q1–Q3)	3.034 [2.750–3.200]	3.059 [2.761–2.960]	2.965 [2.735–3.155]	0.148

Results are presented for all included women and per study arm. Categorical variables were compared using Chi square test and continuous variables using Student’s t test

*LAMP* loop mediated amplification, *SOC* standard of care

### Malaria during pregnancy and anaemia

Haemoglobin was measured at each ANC visit and at delivery. Anaemia prevalence was 45.6%. Overall, 40.9% of women presented with mild anaemia and 4.7% presented with moderate anaemia. These proportions were similar in the LAMP and the SOC arm (45.5% vs. 46.0%). The prevalence of anaemia for women positive by LAMP technology was 66.7% (6/9) (among which 66.7% (4/6) displayed mild anaemia and 33.3% (2/6) displayed moderate anaemia). Overall, LAMP positive patients had an average increase of haemoglobin of 0.9 g/dL between the initial result and delivery. Improved neonatal haemoglobin was observed in the LAMP (13.1 g/dL) versus the SOC (12.8 g/dL) (p = 0.024) arm.

### Performance characteristics of RDT and microscopy versus LAMP as a reference method

The respective sensitivity, specificity, positive predictive value and negative predictive values of RDT, microscopy and LAMP were compared. Microscopy was performed on 585 samples, RDTs on 585 samples and Illumigene LAMP on 435 samples. Analytical performances of RDT and microscopy were evaluated on the 435 samples using LAMP as a reference. Microscopy sensitivity was 55.6% and specificity 100% (Table 3). RDT sensitivity was 66.7% and specificity 100%.

**Table 3:** Performance of rapid diagnostic tests (RDTs) and microscopy using LAMP as a gold standard (n = 435 samples)

LAMP	Microscopy		Total
	Positive	Negative	
Positive	5	4	9
Negative	0	426	426
Total	5	430	435
Sensitivity	55.6% (95% CI 21.10–86.30)		
Specificity	100.00% (95% CI 99.14–100.00)		

LAMP	RDT		Total
	Positive	Negative	
Positive	6	3	9
Negative	0	426	426
Total	6	429	435
Sensitivity	66.7% (95% CI 29.93–92.51)		
Specificity	100.00% (95% CI 99.14–100.00)		

Data is presented as percentages with 95% confidence interval

## Discussion

A longitudinal study was conducted in the Kafa Zone, Ethiopia and included 199 women at the first or second trimester of pregnancy. Women were allocated to either the standard of care (detection by microscopy/RDT) of *Plasmodium* spp. infection or to the LAMP arm. The study population was mainly living in rural areas with a limited education level. Use of malaria prophylactic measures such as ITN was low. Overall, the population is representative of the most vulnerable to MiP and comparable to other recent studies [28, 29]. However, the study population was older (average 26 years) compared to the above mentioned studies (respectively 21 years [29] and 24 years [28]). The retention rate in the study was excellent with only six (3%) pregnant women lost to follow-up.

Cross-sectional studies in the general population from the same area reported a prevalence of *Plasmodium* spp. infection from 5.2% [30] to 11.5% [31] and even 61.6% [32]. Golassa et al. evaluated the prevalence at 3.7% based on microscopy, while the sub-microscopic infection prevalence reached 19.2% [33]. However, the study population is different from our study which focus on pregnant women during ANC visits. The low prevalence of malaria found in this study may also be related to the low peripheral parasitaemia associated with pregnancy. Importantly, immunity may partially control *Plasmodium* infection and hence increase the possibility of sub-microscopic parasitaemia during MiP [4, 5, 34]. The higher sensitivity of LAMP compared to RDT and microscopy for MiP was also reported in a cross-sectional study in Colombia [35].

Regarding the prevalence of anaemia, the proportion of women presenting with anaemia was higher in LAMP-positive patients compared to uninfected patients. This increase may be a reflection of *Plasmodium* infection. Importantly, women who tested positive for *Plasmodium* by LAMP and referred for treatment displayed an increase in maternal and neonatal haemoglobin level at delivery compared to the SOC. Anaemia is, however, multifactorial and nutritional status, deficiencies, and helminths infection are key confounders. For example, a study conducted in 2012 in the Gondar region, Ethiopia, showed that more than half of the study population was positive for soil-transmitted helminths [31].

LBW is an adverse birth outcome associated with MiP that impacts newborn growth and development. A higher proportion of LBW was observed in the SOC arm compared to the LAMP arm. MiP is a key player in fetal growth restriction and subsequent birth outcome [36–38]. The absence of LBW in the LAMP arm may reflect the benefits of LAMP testing of asymptomatic MiP and subsequent treatment. This result requires further investigation in a larger cohort with appropriate controls. Both *P. falciparum* and *P. vivax* are endemic in the study zone. RDT performance for *P. vivax* detection is lower than for *P. falciparum* [39–44]*. Plasmodium vivax* potential to negatively impact pregnancy has been shown [45, 46]. Microscopy and RDT showed a low sensitivity compared to LAMP as reported in previous studies [32, 33, 47]. Further study is required to evaluate the impact of ultra-sensitive LAMP methods based on RNA detection [48]. Nonetheless, this work shows the superiority of LAMP for MiP diagnosis. However, current commercially available LAMP technology devices and reagents are expensive limiting their use in low and middle-income countries. Endemic malaria areas may greatly benefit from LAMP technology but MiP screening strategies can only be implemented if affordable LAMP test become available.

The results presented here have several limitations. No gold standard (such as PCR or RT-PCR) was used. Also, women were arbitrarily allocated to each arm in a pre-determined ratio which may introduce bias. The sample size resulted in a limited number of positive malaria cases limiting the statistical power of the study. Ideally, a comprehensive study for the comparison of IPT and LAMP-based screening of asymptomatic patients would permit a clearer understanding of this strategy. Furthermore, LAMP cannot distinguish active infection from persistent nucleic acid and thus may overcall clinical disease [40, 49, 50]. The study also does not directly address the impact of gametocytaemia as a source of transmission during MiP. In addition, the study design did not permit an analysis of the value of LAMP in symptomatic versus asymptomatic women. Finally, the treatment of patients positive by LAMP was performed outside the study and not directly observed and thus benefits in birth weight and neonatal haemoglobin cannot be necessarily attributed to the LAMP test.

Regarding the practicality of LAMP-based screening for MiP detection, this study shows that the implementation of LAMP is feasible in resource-limited settings. Switching strategies from IPT to screening (LAMP) in settings where IPT is already in place may face additional operational and financial difficulties.

## Conclusions

To summarize, a field study was conducted in the Kafa zone in Ethiopia to assess the impact of LAMP detection of MiP. Interestingly, the LAMP-tested arm showed a lower proportion of LBW and improved newborn hemoglobin levels. Pregnant women and their offspring are likely to benefit from more sensitive tools such as LAMP for MiP detection and subsequent treatment during pregnancy. The findings presented here support further studies using highly sensitive tools for rapid on-site detection of asymptomatic and symptomatic MiP as an alternative to microscopy-based screening or systematic administration of IPT.

## Data Availability

The datasets used and/or analysed during the current study are available from the corresponding author on reasonable request.

## Abbreviations

ANC: Antenatal care
HRP-2: Histidine rich protein 2
IPT: Intermittent preventive treatment
LAMP: Loop mediated isothermal Amplification
LBW: Low birth weight
NPV: Negative predictive value
PCR: Polymerase chain reaction
pLDH: *Plasmodium* lactate dehydrogenase
POC: Point of care
PPV: Positive predictive value
RDT: Rapid diagnostic test
Se: Sensitivity
Sp: Specificity
